# The Effect of Channel Layer Thickness on the Performance of GaN HEMTs for RF Applications

**DOI:** 10.3390/mi16010001

**Published:** 2024-12-24

**Authors:** Qian Yu, Sheng Wu, Meng Zhang, Ling Yang, Xu Zou, Hao Lu, Chunzhou Shi, Wenze Gao, Mei Wu, Bin Hou, Gang Qiu, Xiaoning He, Xiaohua Ma, Yue Hao

**Affiliations:** 1 School of Microelectronics, Xidian University, Xi’an 710071, China; 2 ZTE Corporation, Shenzhen 518057, China; 3 Key Laboratory of Wide Band-Gap Semiconductor Materials and Devices, School of Microelectronics, Xidian University, Xi’an 710071, China; 4 Shaanxi Semiconductor Industry Association, Xi’an 710065, China

**Keywords:** GaN, high electron mobility transistors, Fe-doped, UID-GaN, RF peoformance

## Abstract

In this paper, AlGaN/GaN high electron mobility transistors (HEMTs) with different thicknesses of unintentional doping GaN (UID-GaN) channels were compared and discussed. In order to discuss the effect of different thicknesses of the UID-GaN layer on iron-doped tails, both AlGaN/GaN HEMTs share the same 200 nm GaN buffer layer with an Fe-doped concentration of 8 × 10^17^ cm^−3^. Due to the different thicknesses of the UID-GaN layer, the concentration of Fe trails reaching the two-dimensional electron gas (2DEG) varies. The breakdown voltage (Vbr) increases with the high concentration of Fe-doped in GaN buffer layer. However, the mobility of the low concentration of the Fe-doped tail is higher than that of the high concentration of the Fe-doped tail. Therefore, the effect of different thicknesses of UID-GaN on the DC and radio frequency (RF) performance of the device needs to be verified. It provides a reference to the epitaxial design for high-performance GaN HEMTs.

## 1. Introduction

In the past decade, Gallium Nitride (GaN) high electron mobility transistors (HEMTs) have received attention because of the material properties such as high breakdown voltage, high mobility, and high-temperature resistance [1,2,3,4]. Through the development in recent years, GaN HEMTs have been successfully applied to 5G base stations and mobile terminals [5]. To meet the high power requirements of applications, the breakdown voltage (*V*_BR_) and saturation current (*I*_d,max_) of GaN HEMTs need to be improved. Therefore, a high-resistance GaN buffer layer is needed. The Fe-doped GaN buffer layer is usually used for the radio frequency (RF) GaN HEMTs because the C-doped buffer layer results in serious current collapse (CC) [6,7]. Due to the memory effect of Fe doping, the Fe-doped tail will affect the RF performance of GaN HEMTs [8]. The influence of Fe-doped tail with different GaN buffer layer thicknesses on GaN HEMTs performance has been reported [9]. However, there is a lack of research on the effect of Fe-doped tail with different thicknesses of UID GaN channel layers.

In order to verify the effect of the thickness of different UID GaN channel layers on the performance of the GaN HEMTs, the barrier layer and buffer layer of the same thickness are designed. The impact of channel thickness with C-doped GaN HEMTs has been reported in [10,11]. The two-dimensional electronic gas (2DEG) of the GaN HEMTs with thin UID-GaN channel layers is closer to the Fe-doped GaN buffer layer. The barrier height of GaN HEMTs with a thin UID-GaN channel layer increases faster than that of GaN HEMTs with a thick UID-GaN channel layer. As a result, the 2DEG confinement of the GaN HEMTs is better [12]. The reason why the *V*_BR_ of the GaN HEMTs with thin UID-GaN channel layers is higher is that the buffer leakage current is lower [13]. However, the concentration of the Fe-doped tail is higher than that of the GaN HEMTs with the thick UID-GaN layer. The high Fe doping concentrations introduce additional impurity scattering [14]. This results in a decrease in the mobility of the GaN HEMTs. The saturation current (*I*_d,max_) decreases due to low mobility [15,16].

To provide a reference for the UID-GaN design of RF GaN HEMTs, AlGaN/GaN HEMTs of 650 nm UID-GaN (Sample A) and 250 nm UID-GaN (Sample B) are designed and tested. The material properties of Sample A and Sample B were tested by the Hall measurement. The mobility of Sample A is higher than that of Sample B. The DC performance is characterized by the output current, transfer, and breakdown voltage. Due to the high mobility of Sample A, the output current and the transconductance (*g*_m_) is higher than that of Sample B. The *V*_BR_ of Sample B is higher than that of Sample A. The small signal characteristics of Sample A and Sample B are tested. Finally, to characterize the capabilities of Sample A and Sample B for mobile terminal and base station applications, the load-pull was tested at 3.6 GHz with the bias drain voltage (*V*_d_) of 15 V/28 V/48 V.

## 2. Device Fabrication

**Figure 1:**
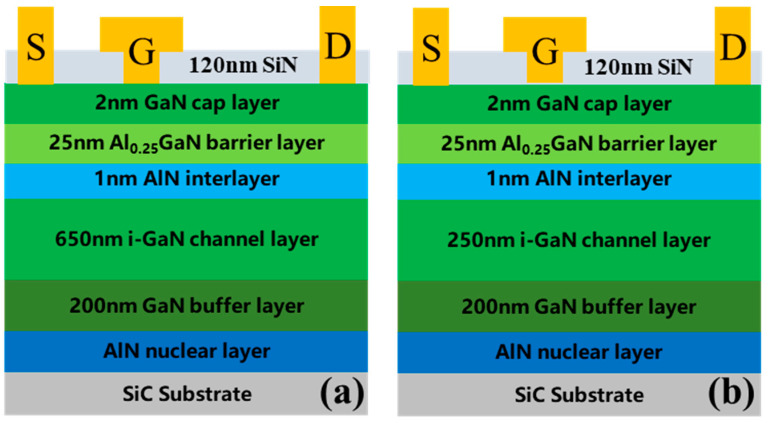
The profiles of (**a**) Sample A and (**b**) Sample B.

The profiles of Sample A and Sample B are shown in Figure 1a,b, respectively. The epitaxial layers of Sample A and Sample B are grown on the 3-inch SiC substrates using metal-organic chemical vapor deposition (MOCVD), including a 200 nm GaN buffer with an Fe-doped concentration of 8 × 10^17^ cm^−3^, a 650 nm unintentionally doped GaN (UID-GaN) channel (250 nm for Sample B), a 1 nm low-temperature AlN interlayer, and a 20 nm Al_0.25_GaN barrier layer. The square resistance of Sample A and Sample B is 275.2 ohm/□ and 349.8 ohm/□, respectively. Due to the low concentration of the Fe-doped tail, the lower square resistance is realized by Sample A. The mobility of Sample A and Sample B is tested by Hall measurement at room temperature. The mobility of Sample A and Sample B is 2183 cm^2^/V·s and 1984 cm^2^/V·s. The carrier density of Sample A and Sample B is 1.04 × 10^13^ cm^−2^ and 8.61 × 10^12^ cm^−2^, respectively.

**Figure 2:**
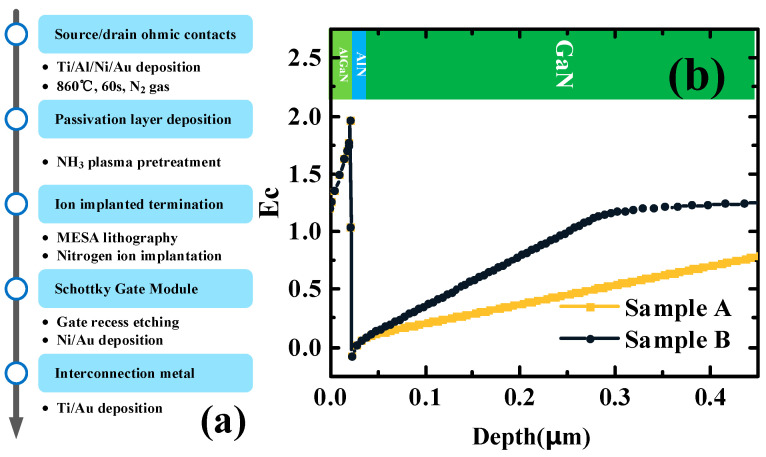
(**a**) The schematic diagram of the GaN HEMTs process flow. (**b**) The energy band diagram of Sample A and Sample B.

The first step of Sample A and Sample B fabrication is the deposition of ohmic metal, Ti/Al/Ni/Au. Next, the ohmic contact of the GaN HEMTs is achieved by annealing at 860 °C for 60 s in the N_2_ ambiance. To achieve the electrical isolation of the GaN HEMTs, the wafer is treated by nitrogen ion implantation. The dosage of the nitrogen ion implantation is 1E15 at/cm^2^. The energy of the nitrogen ion implantation is 200 keV. To reduce CC, a 120 nm SiN_X_ passivation layer is grown using plasma-enhanced chemical vapor deposition (PECVD) at 250 °C. Then, the gate window is defined by lithography and CF_4_-based plasma etching. The gate length (*L*_g_) is 0.5 μm. For the T-type gate, the new step of lithography is used. The gate cap is 1.3 μm. The Ni/Au metal stack is deposited for the gate Schottky contact. Next, lithography is used to achieve the patterns of the interconnections. The final step of the process is to deposit Ti/Au as the interconnection of the GaN HEMTs. The gate-source spacing (*L*_gs_) and gate-drain spacing (*L*_gd_) of GaN HEMTs are 1.5 μm and 3 μm, respectively. The schematic diagram of the process flow is shown in Figure 2a.

The band diagrams of Sample A and Sample B are shown in the Figure 2b. The results are simulated by using the 1D Schrödinger-Poisson solver [17]. The energy level of Fe doping is defined as an acceptor level at 0.5 eV [14]. It can be seen that the barrier increases more slowly in Sample A than in Sample B because the Fe-doped buffer layer is farther away from the 2DEG.

## 3. Results and Discussion

**Figure 3:**
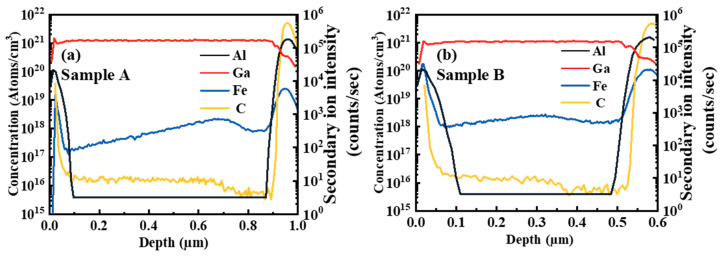
The Secondary Ion Mass Spectrometry of (**a**) Sample A and (**b**) Sample B.

To characterize the distribution of Fe/Al/Ga/C elements along with the height of GaN HEMTs, the Secondary Ion Mass Spectrometry (SIMS) is tested. The results of Sample A and Sample B are shown in Figure 3a,b, respectively. For Sample A, the concentration of the Fe-doped tail reached 2DEG is 2 × 10^17^ cm^−3^. The concentration of the Fe-doped tail of Sample B is 1 × 10^18^ cm^−3^. The concentration distribution of Al, Ga, and C atoms is the same for Sample A and Sample B. The reason why the content of the Al is lower than that of the Ga is that the Al component in the barrier layer is 0.25.

**Figure 4:**
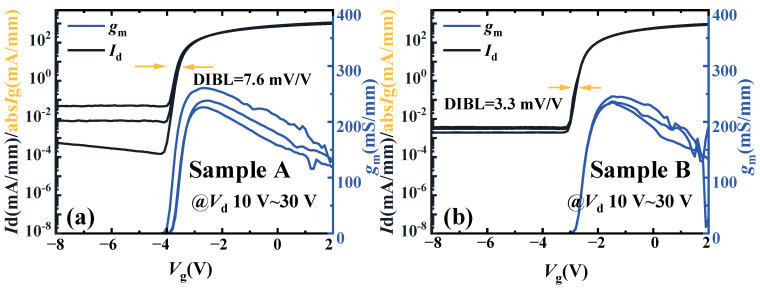
The transfer curves of the (**a**) Sample A and (**b**) Sample B.

The transfer curves of Sample A and Sample B are shown in the Figure 4a,b. The bias drain voltage is 10 V, 20 V, and 30 V. The Drain-Induced Barrier Lowering (DIBL) values of Sample A and B are 7.6 mV/V and 3.3 mV/V, respectively. The abs*I*_g_ is the absolute value of the gate current. As shown in Figure 4, the threshold voltages (*V*_th_) of Sample A and Sample B are −4 V and −2.9 V, respectively. Due to the higher carrier concentration of Sample A, the *V*_th_ of Sample A is more negative than that of Sample B. The peak transconductance (*g*_m,max_) of Sample A is 260 mS/mm at the gate voltage (*V*_g_) = −2.7 V. The *g*_m,max_ of Sample B is 249 mS/mm at the −1 V of *V*_g_. The reason why the *g*_m,max_ of Sample A is higher than that of Sample B is that the mobility of Sample A is higher. The subthreshold swing (SS) of Sample A is 150 mV/dec, which is lower than the 240 mV/dec of Sample B. The DIBL values of Sample A and Sample B are 7.6 mV/V and 3.3 mV/V, respectively.

**Figure 5:**
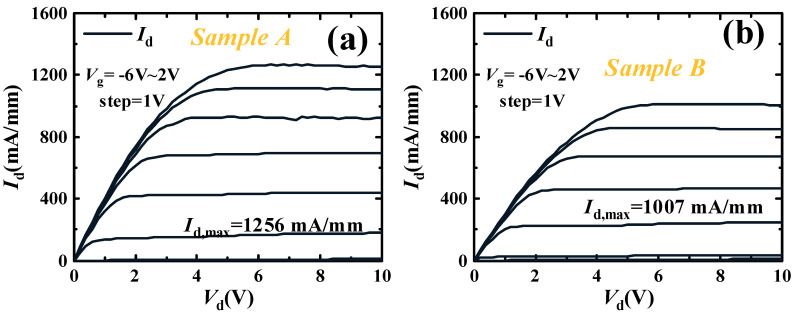
The curves of output current for (**a**) Sample A and (**b**) Sample B.

In Figure 5, the output characteristics of Sample A and Sample B are tested, the *V*_g_ ranges from −6 V to 2 V, and the step is 1 V. The *V*_d_ ranges from 0 V to 10 V. The saturation current (*I*_d,max_) of Sample A is 1256 mA/mm, which is higher than the 1007 mA/mm of Sample B. Due to the influence of the Fe-doped tail, the mobility and carrier concentration of Sample A are higher than those of Sample B. Thus, the *I*_d,max_ of Sample A is higher than that of Sample B. According to the formula for the maximum output power (*P*_max_) of the field-effect transistor (FET), the high *g*_m,max_ and *I*_d,max_ will result in high RF characteristics and output power density. The formula is as follows:

(1) $$P_{\max}=\frac{1}{8}(V_{\mathrm{br}}-V_{\mathrm{knee}})I_{\max}$$

where the *V*_knee_ is the Knee voltage, and the *V*_br_ is the breakdown voltage. The *V*_knee_ is defined as the voltage corresponding to the saturation current at 80%. The *V*_knee_ of Sample A and Sample B is 3.1 V and 3.2 V, respectively. In this regard, the GaN HEMTs with a thick UID-GaN channel layer are more suitable for high-performance RF applications.

**Figure 6:**
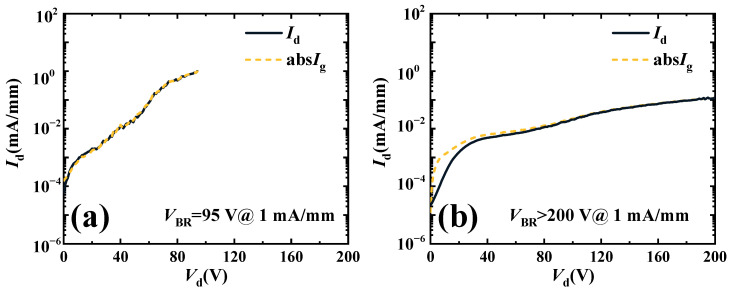
The breakdown of the characteristics of (**a**) Sample A and (**b**) Sample B.

According to Formula (1), the *P*_max_ is not only related to *I*_d,max_, but also to *V*_br_. As shown in Figure 6, the *V*_br_ values of Sample A and Sample B are tested. The *V*_br_ standard is defined as 1 mA/mm. The *V*_br_ of Sample A is 95 V. The *V*_br_ of Sample B is greater than 200 V. The concentration of Fe doping in the buffer of Sample B is higher than that of Sample A. It can be seen that from Figure 6b, the drain leakage current of Sample B is lower than that of Sample A. The drain leakage current of Sample B increases more slowly with leakage voltage than that of Sample A. Thus, the *V*_br_ of Sample B is higher than that of Sample A.

**Figure 7:**
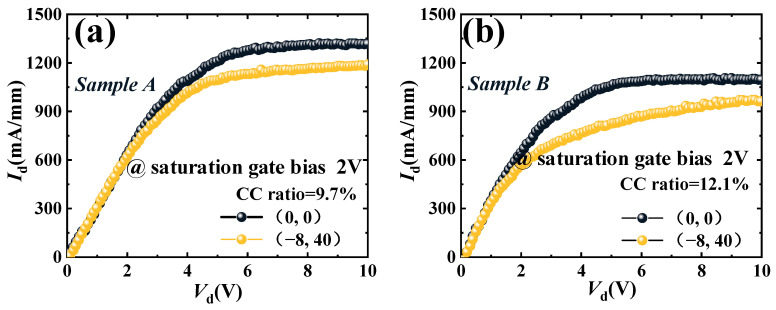
The pulse test curves for Samples A and B, under (0, 0) and (−8, 40).

In order to compare the CC of Sample A and Sample B, the output pulse of Sample A and Sample B are tested under the pinched-off bias voltage 0 V (−8 V) of the quiescent gate bias (*V*_GS,Q_) and 0 V (40 V) of the quiescent drain bias (*V*_DS,Q_). The pulse width of the test is 500 ns, and the period is 1 ms. As shown in Figure 7, the CC ratio is defined as the difference between the current in the saturation zone divided by the saturation current of (0, 0). The CC ratio of Sample A and Sample B is 9.7% and 12.1%, respectively. Due to the high concentration of Fe doping, the doping defect density of Sample B is higher than that of Sample A. Therefore, in the pulse test, the carriers in the channel of Sample B are more likely to be trapped [14,17,18]. The CC ratio of Sample A is lower than that of Sample B. Both the output power density (*P*_out_) and the power-added efficiency (PAE) of the GaN HEMTs are affected by the CC [19].

**Figure 8:**
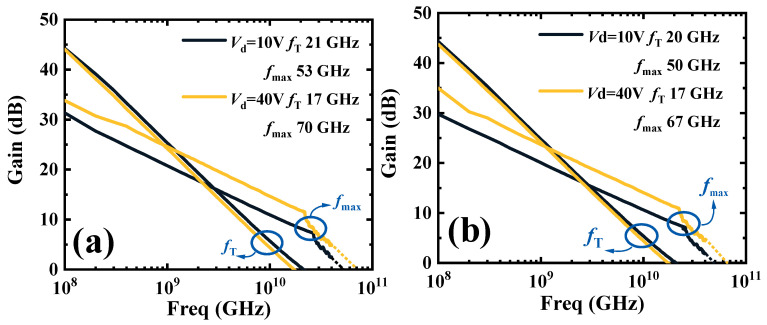
The small signal characteristics of (**a**) Sample A and (**b**) Sample B under the 10 V and 40 V of *V*_d_.

The small signal characteristics of Sample A and Sample B are shown in Figure 8. The drain voltages of the test are 10 V and 40 V. The gate voltages of Sample A and Sample B are −2.8 V and −0.9 V, respectively. This corresponds to the gate voltage of the *g*_m,max_. The current gain cut-off frequency (*f*_T_) and the maximum oscillation frequency (*f*_max_) are obtained by linear extrapolation (−20 dB/decade) of current gain (h_21_) and maximum stable gain (MSG), respectively. At *V*_d_ of 10 V, the *f*_T_ and *f*_max_ of Sample A are 21 GHz and 53 GHz, and those of Sample B are 20 GHz and 50 GHz. At *V*_d_ of 40 V, the *f*_T_ and *f*_max_ of Sample A are 17 GHz and 70 GHz, and those of Sample B are 17 GHz and 67 GHz. Due to the higher *g*_m,max_ of Sample A, the *f*_T_ of Sample A is higher than that of Sample B. Then, the higher *f*_T_ results in a higher *f*_max_. The reason why *f*_T_ decreases with *V*_d_ is that *g*_m_ decreases as *V*_d_ increases [20]. This confirms that better RF gain characteristics are realized by Sample A.

**Figure 9:**
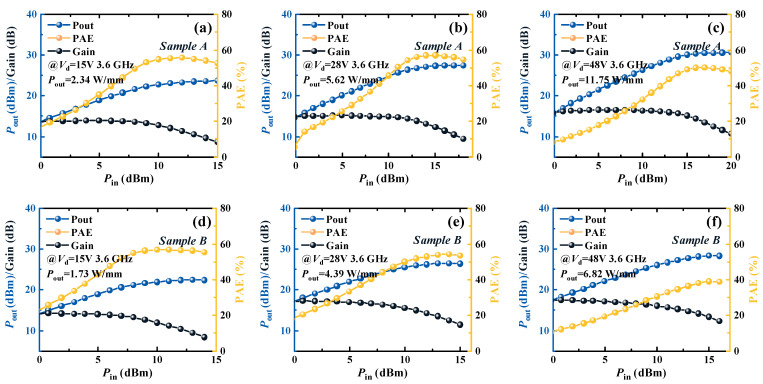
The load-pull of Sample A and Sample B. The (**a**–**c**) are the load-pull under 15 V, 28 V, and 48 V of the *V*_d_ for Sample A, respectively. The (**d**–**f**) are the load-pull under 15 V, 28 V, and 48 V of the *V*_d_ for Sample B, respectively.

The load-pull measurement of Sample A and Sample B is measured at 3.6 GHz using the Maruy. The impedance matching point of the test is matched to the maximum PAE point. The 2nd and 3rd harmonic terminations are not used in the load-pull measurement. To verify the capabilities of Sample A and Sample B for RF mobile terminals, the load pull was tested under the *V*_d_ of 15 V, 28 V, and 48 V shown in Figure 9. The *P*_out_ and PAE of Sample A are 2.34 W/mm and 56%, and those of Sample B are 55% and 1.73 W/mm. At *V*_d_ of 15 V, the PAE of Sample A and Sample B are the same. Sample A has a higher *I*_d,max_, and the gain of Sample A is higher than Sample B. Thus, the *P*_out_ of Sample A is higher than that of Sample B. For base station applications, the load-pull measurement is also tested under the *V*_d_ of 28 V and 48 V. The PAEs of 28 V and 48 V of Sample A are 57% and 50%, respectively. The PAEs of 28 V and 48 V of Sample B are 54% and 40%, respectively. The *P*_out_ of 28 V and 48 V of Sample A are 5.62 W/mm and 11.75 W/mm, and those of Sample B are 4.39 W/mm and 6.82 W/mm. As the *V*_d_ increases, more traps of Sample B are activated. This results in serious CC. Thus, the load-pull characteristics of Sample A are better than those of Sample B under the high *V*_d_. In conclusion, Sample A is more suitable for the RF mobile terminals and base station applications than Sample B.

## 4. Conclusions

In this paper, the AlGaN/GaN HEMTs with different UID-GaN thicknesses are fabricated and compared. In order to verify the effect of Fe-doped tails on the performance of the GaN HEMTs with different UID-GaN thicknesses, the DC and RF performance of Sample A and Sample B is evaluated. For the DC performance, the *g*_m,max_ and *I*_d,max_ of Sample A are higher than those of Sample B. However, the *V*_br_ of Sample B is higher than that of Sample A. For the RF characteristics, the *f*_T_ and *f*_max_ of Sample A are higher than those of Sample B at the *V*_d_ of 10 V and 40 V. Due to the serious CC, the PAE and *P*_out_ of Sample B are lower than those of Sample A. In conclusion, Sample A is more suitable for the RF mobile terminals and base station applications than Sample B.

## Data Availability

The original contributions presented in the study are included in the article, further inquiries can be directed to the corresponding authors.
